# Demographics, Clinical Presentation and Outcome of Metapneumovirus Infection in Adults: A Case Series Analysis at Scarborough General Hospital, United Kingdom

**DOI:** 10.7759/cureus.73292

**Published:** 2024-11-08

**Authors:** Amala Khan, Vishesh Khanna, Kausik Majumdar

**Affiliations:** 1 Emergency Medicine, York Hospital, York, GBR; 2 Internal Medicine, Scarborough Hospital, Scarborough, GBR; 3 Geriatric Medicine, Scarborough Hospital, Scarborough, GBR

**Keywords:** acute respiratory infection, adults, demographics, hmpv, human metapneumovirus, treatment, viral pneumonia

## Abstract

Introduction: Human metapneumovirus (hMPV) was first discovered in 2001 in Netherlands as a leading cause of respiratory infections. hMPV infection is more common in kids, elderly (age ≥ 65) and immuno-compromised adults. Treatment is mainly symptomatic.

Methodology: We collected retrospective data from 31-8-2022 to 01-09-2023 from Microbiology for patients who tested positive for hMPV by polymerase chain reaction (PCR). Only patients aged 18 years and above and admitted to Scarborough General Hospital (SGH) were included in the study.

Results: Total patients who tested positive were 38, out of which 73% (n=24) of patients were ≥ 65 years of age. 76.3% (n=29) of these adults were living in their own residence and 53% (n=20) patients never smoked. The most common presentation of these patients was shortness of breath and cough. Fifty-eight percent (n=22) patients had no radiological findings and 74% (n=28) had raised C-reactive protein (CRP). hMPV management was analyzed based on six modalities, we found out that 76% (n=29) patients received antibiotics, 47% (n=18) received nebulizers, 45% (n=17) required oxygen, 37% (n=14) were treated with steroids, 21% (n=8) patients were given inhalers and only one received antivirals. Majority of the patients were discharged and 13% (n=5) of patients died during their inpatient stay. All the deceased patients were aged 65 and above and 80% (n=4) of deceased (n=5) had pre-existing co-morbidities or other acute diagnoses at admission.

Conclusion: The patients who tested positive for hMPV were mostly aged ≥ 65 years, 76.3% (n=28) were from personal residence and there was no association of smoking history with hMPV infection. Patients who tested positive for hMPV would mostly present with flu-like symptoms with raised CRP and no radiological manifestation. All these patients were managed conservatively with antibiotics, nebulizers, oxygen, inhalers and antivirals (only one patient). Most of the patients were discharged home and five died during the inpatient stay, all of them were >65 of age and 80% had pre-existing co-morbidities and other acute diagnoses at the time of admission. We could not conclude or hypothesize anything due to small sample size.

Limitations: This data was collected over a one-year period only, and the sample size was very small. Another limitation was that we did not follow up patients after discharge.

## Introduction

Human metapneumovirus (hMPV), a leading cause of acute respiratory infection in humans, was first identified in 2001 by scientists in the Netherlands [1-3]. hMPV can cause upper and lower respiratory disease in people of all ages [4,5]. Approximately 90-100% of children are affected by hMPV between the ages of five to 10 as per seroprevalence studies [6-9]. Infection can reoccur within the elderly population and immunocompromised individuals [10,11]. Broader use of molecular diagnostic testing techniques like polymerase chain reaction (PCR) has increased identification and awareness about this virus [1].

hMPV is a negative sense single-stranded ribonucleic acid (RNA) virus, part of the Pneumoviridae family of viruses, similar to respiratory syncytial virus (RSV) [1,3]. It can cause mild upper respiratory tract infection in healthy individuals with symptoms like cough, sore throat and fever [1,10]. hMPV is mostly spread from an infected person to healthy individuals through secretions from coughing and sneezing, close personal contact and fomites [1,12]. Thus, hMPV infection is more common in closed or shared accommodation, as was seen in the respiratory infection outbreak in two skilled nursing facilities in West Virginia and Idaho, during 2011-2012 [13]. Another retrospective survey for three northern hemisphere influenza seasons from 2010 to 2013, which included 590 care homes and 75 outbreaks, showed that 10 outbreaks out of 75 were caused by parainfluenza, hMPV or RSV [14].

hMPV circulates in distinct annual seasons. hMPV circulation begins in winter and lasts until or through spring [1,15,16]. hMPV, RSV, and influenza can circulate simultaneously during the respiratory virus season [1]. In a study undertaken in Tennessee, during three influenza seasons from 2006 to 2009, it was observed that in adults aged ≥ 50 years, hospitalization rates for RSV and hMPV were similar to those associated with Influenza [17]. Another study including six hospitals in North Dakota was conducted after a respiratory illness breakout from 31 July 2015 to 26 May 2016 and showed 27 adults were laboratory-confirmed hMPV positive, out of which three patients died [18]. In 2019, a global systematic analysis on monthly activity patterns of different viruses that included 246 sites, out of which 65 were hMPV affected, showed that hMPV epidemics occurred in late winter and spring in most temperate sites. However, the timing of epidemics was more diverse in the tropics [19].

Currently, there is no specific antiviral therapy to treat hMPV and no vaccine to prevent hMPV. Medical management is supportive, as infection mostly resolves on its own. Treatment is mostly aimed at easing symptoms like cough, fever and shortness of breath. Patients with more severe symptoms may require oxygen support and oral or inhaled corticosteroids [1,10]. Administration of oral or aerosolized ribavirin with or without polyclonal intravenous immunoglobulins (IVIGs) has been advocated for the treatment of severe hMPV infections and in high-risk patients [2,20].

Aims

The aims of this study were 1) To identify the hMPV cases at Scarborough General Hospital (SGH) in one year; 2) To analyze the demographics of hMPV patients; and 3) To survey treatment modalities and patient outcomes.

## Materials and methods

Study design and participants

This was a retrospective study to gather and analyze data of patients diagnosed with hMPV over a specified timeframe of one year, from August 31, 2022, to September 1, 2023. The initial step involved obtaining a list of patients who tested positive for hMPV via PCR testing. This data was sourced from the Microbiology department of SGH.

To ensure a focused and relevant analysis, we set a specific inclusion criterion. Only patients who were aged 18 years and older were considered for the study. Additionally, all included patients were required to have been admitted to SGH during the above-specified timeframe.

Data collection

Data was collected retrospectively for this study by a thorough assessment of electronic patient health records at SGH. Data collection was focused on several critical aspects of health status and management of patients who tested positive for hMPV. Essential demographic information, specifically the age and gender of hMPV-positive patients, was documented. Information regarding patients’ social backgrounds was also recorded. This included smoking history, present or past, and residential status of the patients, focusing on whether they presented to SGH from their own personal residences or care homes/nursing homes.

Any pre-existing medical conditions or co-morbidities of these patients were recorded to establish if there was any common link between hMPV and medical conditions like chronic obstructive pulmonary disease (COPD), asthma or malignancy. Symptoms of hMPV-positive patients at the time of diagnosis were documented, to establish clinical patterns associated with hMPV. Data was collected on laboratory blood test results, particularly infection markers like white cell count (WCC) and C-reactive protein (CRP). Radiological findings from chest X-rays were also noted.

The various treatment modalities like antibiotics, anti-viral drugs, oxygen and nebulizers that were used to manage hMPV-related respiratory symptoms were also recorded. Finally, we noted the final outcome for each patient, whether they were successfully discharged or deceased. For deceased patients, we also documented any other acute diagnosis present during admission. It should be noted that follow-up data on patients after discharge was not collected.

Statistical analysis

All collected data was categorized into discrete, categorical variables, which represent distinct groups or categories rather than continuous measurements. As a result, the data was presented as frequency counts (n) and corresponding percentages (%).

Given that the sample size was relatively small, we opted for a straightforward data compilation approach using basic Microsoft Excel (Microsoft, Redmond, WA, USA). We effectively summarized the data, facilitating a clear presentation of findings through tables and charts that outline the frequency of various demographic and clinical characteristics.

Due to the limited sample size, we did not conduct any advanced statistical analysis. Instead, the focus was on descriptive statistics to provide a foundational understanding of the patient population and their management and outcomes with hMPV.

## Results

As per our findings, a total number of 38 patients were found to have tested positive for hMPV in the one-year period. Out of these 38 patients, there were 50% males (n=19) and 50% females (n=19). Age distribution findings showed that there were 37% (n=14) patients less than 65 years old with hMPV positive and there were 73% (n=24) patients who were aged 65 and above. 76.3% (n=29) of patients who tested positive for hMPV were living in their personal residence, 18.4% (n=7) had no living condition documented and 5.2% (n=2) were from a care home/nursing home (Table 1).

**Table 1:** Residential Status and Outcomes

Residential Status	Number of Patients (n)
Care/Nursing home	2
Discharged	2
Residential Status not recorded	7
Discharged	7
Personal residence	29
Deceased	5
Discharged	24
Grand Total	38

**Figure 1:**
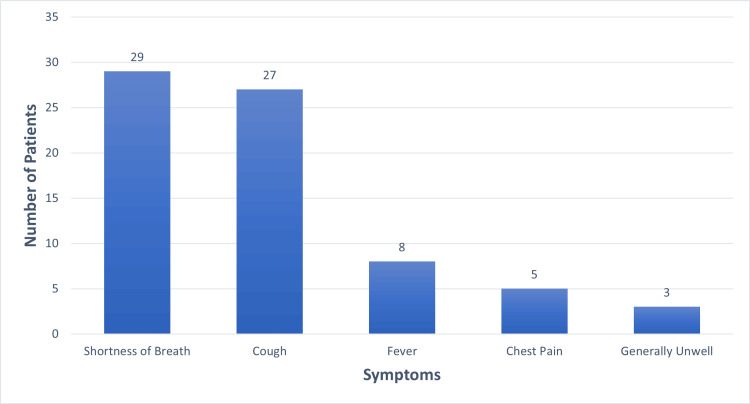
Presenting Symptoms

76.3% (n=29) patients reported shortness of breath, 71% (n=27) patients reported cough, 21% (n=8) patients reported fever and 5% (n=5) reported chest pain (Figure 1). Only 8% (n=3) of these 38 individuals were current smokers, 31% (n=12) were ex-smokers, 53% (n=20) never smoked and 8% (n=3) did not have any smoking history data recorded.

**Figure 2:**
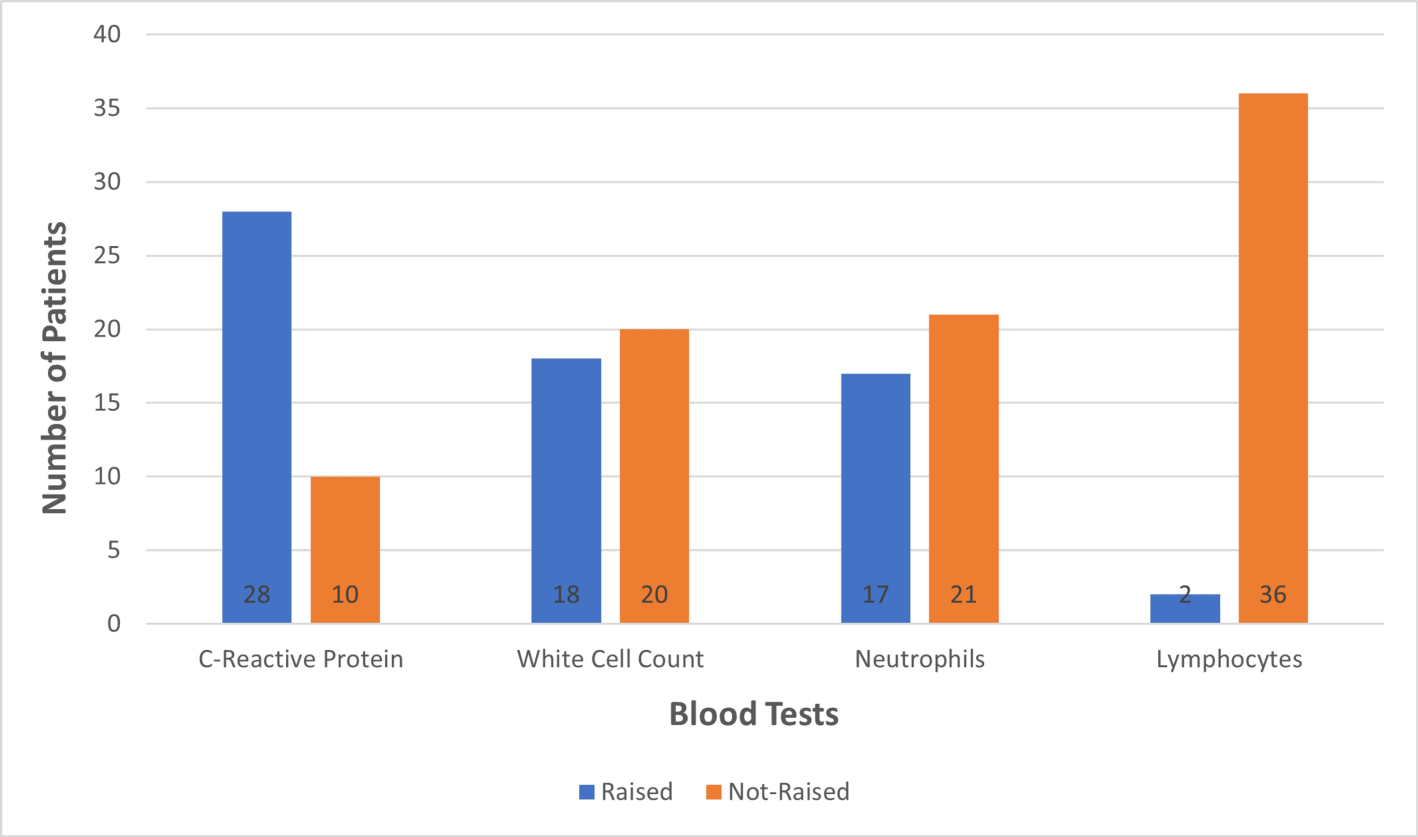
Blood Test Results

Radiological findings included no changes on X-ray for 58% of these individuals (n=22), 29% (n=11) patients had unilateral changes (i.e. consolidation or haziness) and 13% (n=5) patients had bilateral changes. After analyzing blood test results, we found 74% (n=28) of patients had raised CRP. Forty-seven percent (n=18) of patients had raised WCC, predominantly neutrophilia (Figure 2).

**Figure 3:**
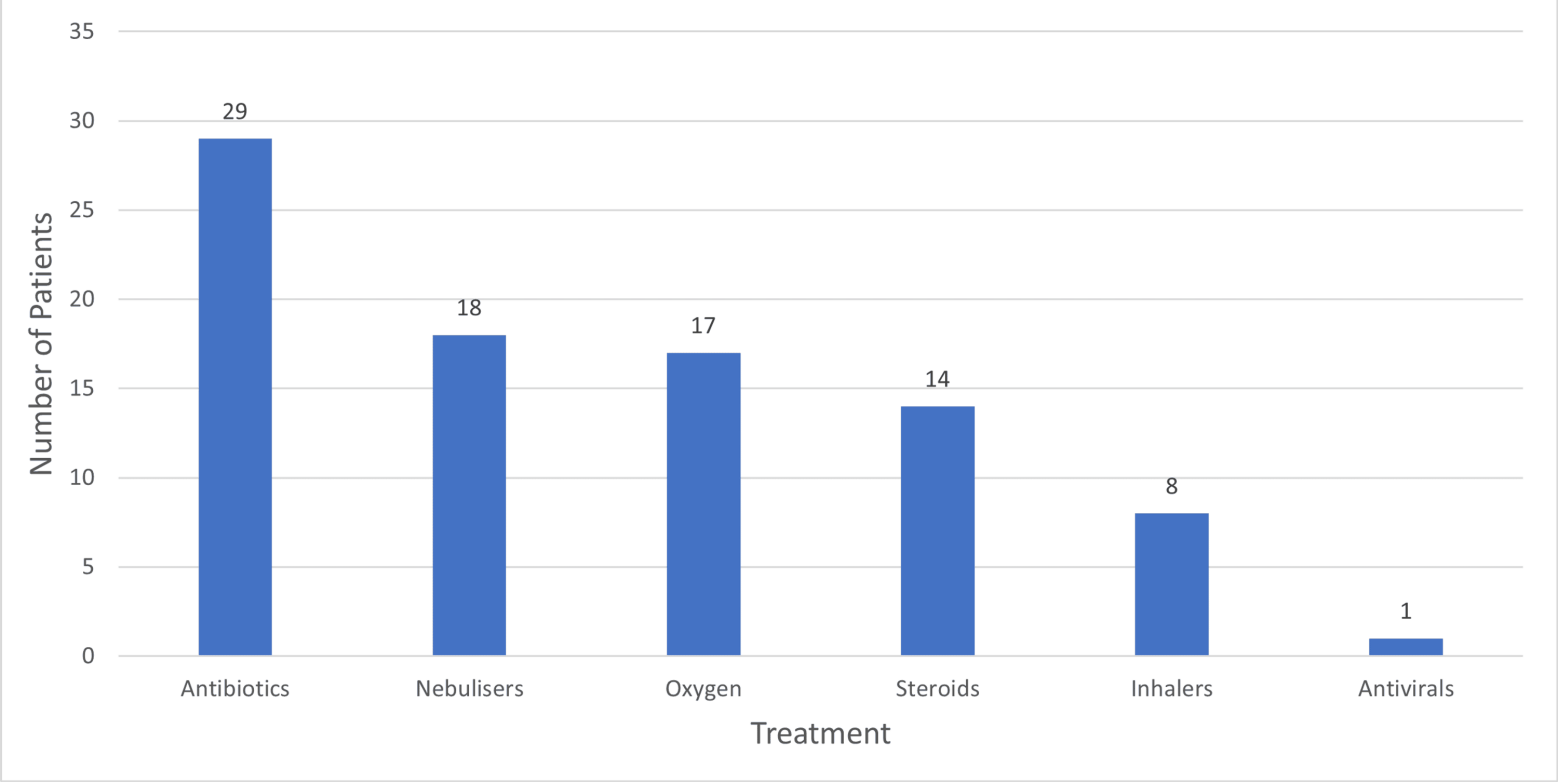
Treatment modalities

hMPV management was analyzed based on six modalities (Figure 3). We found out that 76% (n=29) patients received antibiotics, 47% (n=18) received nebulizers, 45% (n=17) required oxygen, 37% (n=14) were given steroids, 21% (n=8) patients were given inhalers and only 2.6% (n=1) received antivirals (Figure 3). Records showed that 87% (n=33) were discharged and 13% (n=5) patients died during their inpatient stay.

Among the deceased patients, Patient 1 had hypertension, transitional cell carcinoma of bladder, bronchiectasis and previous pulmonary embolism; Patient 2 had COPD, osteoarthritis, Raynaud’s syndrome; Patient 3 had diabetes mellitus and prostate cancer; Patient 4 had hypertension; Patient 5 had no co-morbidities at all. Four out of the five deceased patients also had additional acute diagnoses during admission, i.e. Patient 1 had infective exacerbation of COPD, cryptogenic pneumonitis, and respiratory syncytial virus infection; Patient 2 had bronchopneumonia; Patient 3 had decompensated heart failure; Patient 4 had base of skull fracture and aspiration pneumonia; but Patient 5 had no associated diagnosis during admission.

## Discussion

hMPV is a significant pathogen responsible for respiratory infections, mainly in children, the elderly, and immunocompromised individuals [10]. Symptoms can range from mild cold-like symptoms to severe pneumonia, and it often mimics symptoms associated with other respiratory viruses like influenza and RSV [15]. The virus spreads through respiratory droplets. There's no specific antiviral treatment, and generally supportive care is recommended. Vaccines are still under research and development [6,20]. Therefore, understanding hMPV's epidemiology and clinical impact is crucial for public health management.

This data from Scarborough General Hospital highlights the importance of considering hMPV as a potential cause of respiratory infections in adults. By conducting extended viral screening, the study revealed that hMPV may be more prevalent than currently recognized in existing literature, particularly in adult patients whose symptoms are otherwise unexplained.

Our aim was to look into the demographics of the patients who tested positive for hMPV, and we found an equal distribution of hMPV infection between males and females suggesting that the virus may affect both genders equally. The higher prevalence among elderly patients (age >=65) aligns with existing literature indicating that the elderly population is more susceptible to respiratory infections, possibly due to age-related decline in immunity. Majority of the patients were admitted from personal residences, which suggested that patients living in their own house/residence might also be equally prone to the infection. This is in contradiction to the common assumption that people living in assisted living are more prone to viral infections. The lack of correlation between smoking history and infection rates suggested that smoking may not be a key risk factor for hMPV infection.

The clinical presentation of hMPV is similar to seasonal flu symptoms as it is a virus affecting the respiratory system. The most common presenting symptom was shortness of breath and cough, followed by fever and chest pain. The analysis of blood test results revealed that CRP was raised in the majority of hMPV-positive patients whereas the rest of the infection markers were inconclusive. WCC was raised in 47% (n=18) patients, but most of them had mild elevation.

The patients included in this case series were also managed for other co-morbidities and co-diagnosis present at the time of admission. We only analyzed the treatment that was relevant to respiratory tract infections. This included six modalities; i.e. antibiotics, antivirals, nebulizers, oxygen, inhalers, and steroids. The data showed that most of the patients received antibiotics. Patients with more severe symptoms received nebulizers, oxygen support and steroids. Only one patient was recorded to have been given anti-viral drugs (oseltamivir). It should be noted that this patient did not test positive for any other viral infection and the outcome was recorded as discharged.

Out of 38 patients who tested positive for hMPV infection, the majority were successfully discharged. Among the deceased patients, all were aged 65 and above. There were no common co-morbidities or common pre-existing illnesses found among them. One patient (Patient 5) among the deceased had no past medical history/co-morbidities. We also explored associated acute diagnoses that these deceased patients had at the time of admission and three patients had illnesses that could have contributed to death. Two patients among the deceased had no other acute diagnosis that could have contributed to death (Patient 4 had bronchopneumonia; likely due to hMPV virus, and Patient 5 had no associated acute diagnosis).

Limitations

Our case series was limited because we had data from one year only. Given the fact that we do not have enough existing literature on hMPV, we need data from a longer time frame. Our total sample size was very small to form any hypothesis. Another limitation was that we were not able to follow up patients after discharge, which might have given us false deceased numbers.

## Conclusions

This case series was undertaken to study the demographics and clinical presentation of patients who tested positive for hMPV and to analyze management modalities and outcomes. We observed that majority of patients who tested positive for hMPV were aged >= 65. A large number of patients with positive hMPV tests hailed from personal residence. Shortness of breath was the most common presenting symptom, followed by cough. CRP was the most commonly raised infection marker. A majority of patients received antibiotics; nebulizers, steroids and oxygen therapy were used in more severe cases. Most of the patients were successfully discharged from the hospital. Among the deceased patients, all were aged 65 and above and 80% (n=4) of the deceased individuals (n=5) had pre-existing co-morbidities or other acute diagnoses at admission.

We observed a deviation from the literature in the fact that most of our patients hailed from personal residences. Also, one patient died despite having no co-morbidities or other diagnosis apart from hMPV. This needs to be evaluated on a bigger scale as our data was very limited in terms of quantity.

## References

[1] (2024). About Human Metapneumovirus. https://www.cdc.gov/human-metapneumovirus/about/.

[2] Chen L, Han X, Bai L, Zhang J (2021). Clinical characteristics and outcomes in adult patients hospitalized with influenza, respiratory syncytial virus and human metapneumovirus infections. Expert Rev Anti Infect Ther.

[3] Panda S, Mohakud NK, Pena L, Kumar S (2014). Human metapneumovirus: review of an important respiratory pathogen. Int J Infect Dis.

[4] Walsh EE, Peterson DR, Falsey AR (2008). Human metapneumovirus infections in adults: another piece of the puzzle. Arch Intern Med.

[5] van den Hoogen BG (2007). Respiratory tract infection due to human metapneumovirus among elderly patients. Clin Infect Dis.

[6] Kahn JS (2006). Epidemiology of human metapneumovirus. Clin Microbiol Rev.

[7] Wolf DG, Zakay-Rones Z, Fadeela A, Greenberg D, Dagan R (2003). High seroprevalence of human metapneumovirus among young children in Israel. J Infect Dis.

[8] Ebihara T, Endo R, Kikuta H, Ishiguro N, Yoshioka M, Ma X, Kobayashi K (2003). Seroprevalence of human metapneumovirus in Japan. J Med Virol.

[9] Pavlin JA, Hickey AC, Ulbrandt N (2008). Human metapneumovirus reinfection among children in Thailand determined by ELISA using purified soluble fusion protein. J Infect Dis.

[10] (2024). Human metapneumovirus (hMPV). https://www.lung.org/lung-health-diseases/lung-disease-lookup/human-metapneumovirus-hmpv.

[11] Boivin G, Abed Y, Pelletier G (2002). Virological features and clinical manifestations associated with human metapneumovirus: a new paramyxovirus responsible for acute respiratory-tract infections in all age groups. J Infect Dis.

[12] van den Hoogen BG, van Doornum GJ, Fockens JC (2003). Prevalence and clinical symptoms of human metapneumovirus infection in hospitalized patients. J Infect Dis.

[13] (2013). Outbreaks of human metapneumovirus in two skilled nursing facilities — West Virginia and Idaho, 2011-2012. MMWR Morb Mortal Wkly Rep.

[14] Millership S, Cummins A (2015). Oseltamivir in influenza outbreaks in care homes: challenges and benefits of use in the real world. J Hosp Infect.

[15] (2024). Respiratory syncytial virus (RSV): symptoms, transmission, prevention, treatment. https://www.gov.uk/government/publications/respiratory-syncytial-virus-rsv-symptoms-transmission-prevention-treatment/respiratory-syncytial-virus-rsv-symptoms-transmission-prevention-treatment.

[16] Haynes AK, Fowlkes AL, Schneider E, Mutuc JD, Armstrong GL, Gerber SI (2016). Human metapneumovirus circulation in the United States, 2008 to 2014. Pediatrics.

[17] Widmer K, Zhu Y, Williams JV, Griffin MR, Edwards KM, Talbot HK (2012). Rates of hospitalizations for respiratory syncytial virus, human metapneumovirus, and influenza virus in older adults. J Infect Dis.

[18] Midgley CM, Baber JK, Biggs HM (2017). Notes from the field: severe human metapneumovirus infections — North Dakota, 2016. MMWR Morb Mortal Wkly Rep.

[19] Schuster JE, Williams JV (2014). Human metapneumovirus. Microbiol Spectr.

[20] Li Y, Reeves RM, Wang X (2019). Global patterns in monthly activity of influenza virus, respiratory syncytial virus, parainfluenza virus, and metapneumovirus: a systematic analysis. Lancet Glob Health.

